# Occupational therapy for epidermolysis bullosa: clinical practice guidelines

**DOI:** 10.1186/s13023-019-1059-8

**Published:** 2019-06-07

**Authors:** Jennifer M. Chan, Amy Weisman, Alex King, Susan Maksomski, Carrissa Shotwell, Claire Bailie, Helen Weaver, Rebecca Bodan, Estrella Guerrero, Matija Zmazek, Phuong Khuu

**Affiliations:** 1 Department of Rehabilitation, Lucile Packard Children’s Hospital Stanford, 321 Middlefield Road, Menlo Park, CA 94025 USA; 2 Phoenix Children’s Hospital, Phoenix, USA; 3 Brooklyn Methodist Hospital, Brooklyn, USA; 4 Cincinnati Children’s Hospital Medical Centre, Cincinnati, USA; 5 Sydney Children’s Hospital, Sydney, Australia; 6 Community Project Lead DEBRA UK, Crowthorne, UK; 7 School of Nursing, California State University Fullerton, Fullerton, USA; 8 Community Social worker DEBRA Spain, Marbella, Spain; 9 DEBRA Croatia, Zagreb, Croatia

**Keywords:** Epidermolysis bullosa, Occupational therapy, Clinical practice guideline, Activities of daily living

## Abstract

The purpose of this article is to summarize the Dystrophic Epidermolysis Bullosa Research Association (DEBRA) International evidence-based Clinical Practice Guidelines (CPGs) for the provision of occupational therapy (OT) for children and adults living with inherited epidermolysis bullosa (EB). This is a rare genetic disorder characterized by skin fragility leading to blister formation occurring spontaneously or following minor trauma. Current OT practice for persons with EB is based on anecdotal care, clinical expertise and trial and error with collaboration between caregiver and patient. Intervention based on research is needed to establish a foundation of knowledge to guide international practitioners to create and improve standards of care and to be able to work effectively with those living with the rare diagnosis of this condition.

This CPG was created by an international panel with expertise working with persons with EB. The panel was made up of 11 members including OT’s, a physiotherapist, a medical doctor, social worker, person with EB and a carer of a person with EB. It describes the development of recommendations for 5 outcomes determined by survey of persons with EB, caregivers, and experienced healthcare professionals. The outcomes include independence in activities of daily living (ADL), independence in instrumental ADL, maximization of hand function (non-surgical), fine motor development and retention, and oral feeding skills. The recommendations are supplemented with additional files that include photos and specific examples to further guide occupational therapists or, in situations where an OT is not available, other members of the healthcare team.

As the disorder of EB is rare, evidence-based CPGs are needed to provide a base of knowledge and practice for OTs throughout the world with the goal of providing quality care to patients, while improving their functional independence and quality of life. In addition, this information is valuable as a basis for further research.

**Electronic supplementary material:**

The online version of this article (10.1186/s13023-019-1059-8) contains supplementary material, which is available to authorized users.

## Background

**Fig. 1:**
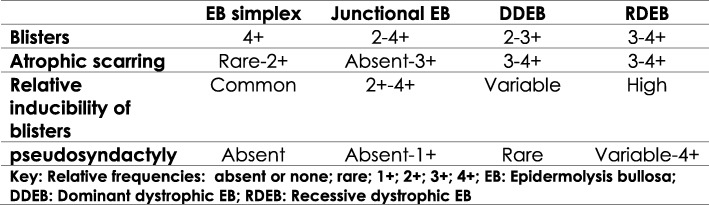
Comparative finding in the major subtypes of EB [1]

Epidermolysis bullosa (EB) is a rare genetic disorder characterized by skin fragility with blister formation occurring spontaneously or following minor trauma such as gentle pressure or friction. It can be broadly divided into four major subtypes: EB simplex (EBS), junctional (JEB), dystrophic (DEB), and Kindler syndrome (KS). EB can affect multiple body systems, particularly the skin. Subtypes are determined by several factors including the level of skin cleavage, phenotype, mode of inheritance, and molecular origin. Considerable variation may exist in disease severity and the natural history of patients within even a single EB subtype or kindred, because of the influence of environmental and/or modifying genetic factors. Generally speaking, EBS encompasses all subtypes of EB with mechanical fragility and blistering confined to the epidermis. JEB includes all subtypes with blister formation within the lamina lucida of the skin basement membrane. DEB patients have blister formation in the uppermost dermis. KS patients have blister formation in multiple levels within or beneath the basement membrane. Figure 1 is an excerpt from the Clinical Findings Table from the latest international consensus meeting on diagnosis and classification of EB in 2014 [1]. It summarizes the relative occurrence of aspects of EB that may affect the function of persons within the various subtypes.

Persons with EB present with a range of disabilities. The more severely affected may experience scarring, fibrosis, and contractures affecting any part of the body. Pain can be both acute and chronic, independence in activities of daily living (ADL) may be significantly limited, and quality of life (QoL) can be affected. There is currently no cure for EB, yet supportive care for symptom relief can be provided through a multidisciplinary team (MDT) approach where available. An occupational therapist (OT) may be an integral part of the MDT.

The practice of OT means the therapeutic use of occupations, including everyday life, activities among individuals and/or groups. The practice of OT supports participation, performance, and function in roles and situations in home, school, work place, community and other settings. OT services are provided for habilitation and rehabilitation. They promote health and wellness to those who have or are at risk for developing an illness, injury, disease, disorder, condition, impairment, disability, activity limitation, or participation restriction [2]. OT addresses the physical, cognitive, psychosocial, sensory-perceptual, and other aspects of performance in a variety of contexts and environments, to support engagement in occupations that affects physical and mental health, and QoL.

Current OT practice with EB patients is based on anecdotal care, clinical expertise and trial and error with collaboration between caregiver and patient. Evidence-based clinical practice guidelines (CPGs) are needed to establish a foundation of knowledge to guide international practitioners to create and improve standards of care and to be able to work effectively with those living with the rare diagnosis of EB.

## Objectives

- To provide the users with information on current best-practices for the provision of OT for people with EB based on a systematic review of evidence. Where possible, the information will be categorized for paediatric and adult patients.
- This information may be applicable for all patients with variations within the subtypes of EB who have limitations in their ADL due to pain, blister formation, and contractures.

### Guideline users

OTs, those living with EB and their carers, rehabilitation practitioners, allied health professionals(AHP), nurses, physiotherapists (PTs), physicians/medical doctors, physician assistants, social workers, educational staff, and employers of persons with EB.

### Target group

These guidelines can be applied to all persons diagnosed with all subtypes of EB who are experiencing limitations.

### Methods used for developing this guideline and formulating the recommendations

In 2016, an international panel of 11 members was co-ordinated through DEBRA International (DI) through a voluntary membership. They represented OT, PT, nursing, social work, dermatology, a parent/care-giver, and person with EB, experts from the United Kingdom (UK), Australia, Croatia, the United States (US) and Spain.

The OT CPG lead (JC) and co-lead (AW) acted as the primary methodologists with consultation from expert researchers. They attended Guideline International Network conference in Philadelphia 2016 for training in Grades of Recommendation Assessment, Development and Evaluation (GRADE) [3] methodology and completed online training in Scottish Intercollegiate Guidelines Network (SIGN) [4] methodology.

In 2017, a scoping survey was created and focused on topics relevant to OTs working with patients with EB in an effort to prioritize the outcomes. The survey was distributed to health care providers working with patients with EB, caregivers, and people living with EB; in the US, UK, Australia, Spain, and Croatia. A total of 33 surveys were collected. This data informed the first meeting to identify the clinical question(s) and outcomes for the CPG. The meeting was attended by 6 panel members physically present and 4 via teleconference.

#### Literature search

**Fig. 2:**
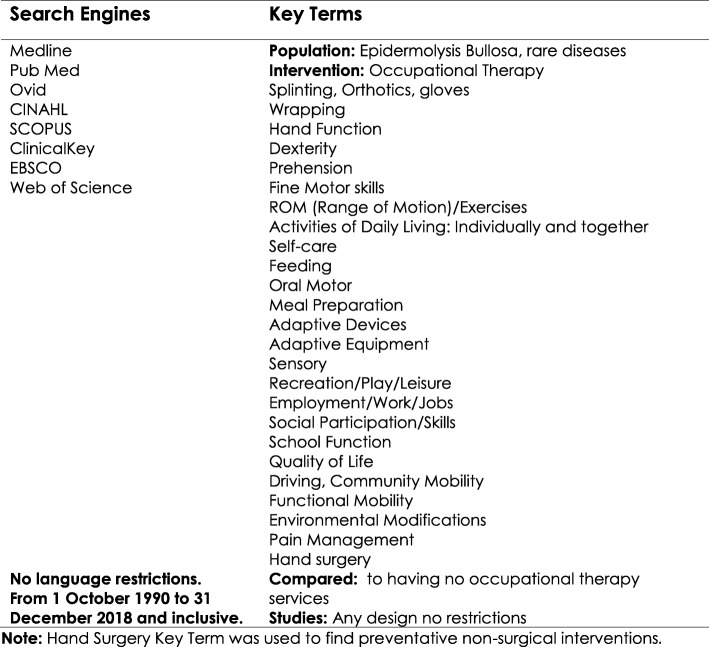
Search Engines, Key Terms, and Criteria

A systematic format was developed to gather literature based on key terms. Search terms were kept broad utilising PICOS. The boolean AND and OR operators were used to combine these terms as appropriate (Fig. 2). Literature searches were conducted by a librarian and 4 panel members through their institutions to access eight electronic search engines listed in Fig. 2. Searches included years October 1990–December 2018 (Fig. 2). The 4 members filtered by title and abstract. Those articles that did not address the identified outcomes and criteria of inclusion were excluded. Articles pertaining to people with EB were prioritized. Due to the paucity of information, a wider search using outcome search terms addressing patients receiving occupational therapy with other chronic conditions was also conducted. Citations from the evidence searches were reviewed by title and abstract for potential inclusion regardless of study design (Fig. 2).

All panel members were trained by the leads for critical appraisal stage which utilized a combination of SIGN [4], GRADE [3], and Critical Appraisal Skills Programme (CASP [5]). All articles were read in full by at least 2 appraisers for consistency, except 2 Spanish articles reviewed by a Spanish speaking panel member. 40 articles were excluded as a result of this process. The remaining articles were categorized based on outcomes with some articles overlapping more than one outcome (Table 2). Summary tables of the outcomes were presented at the final meeting for grading the strength of the recommendations, in Salzburg Austria on 26th September 2017.

In 2018, 15 expert independent referees were invited to review the CPG and they signed a roles and responsibility agreement and CoI forms prior to the anonymised manuscript being circulated. Reviewer comments were taken into consideration for the final version of the OT CPG. On panel agreement all recommendation summaries were circulated to the panel and final feedback was included. Furthermore the Appraisal of Guidelines for Research & Evaluation (AGREE) [6] II tool was consulted to increase the quality of practice guidelines in rare diseases and this CPG acknowledges other guidelines by signposting with the symbol ⇒ through this manuscript.

## Results

The recommendation summary has been grouped by age if the recommendation refers to a specific age level (Table 1). Infant is defined as birth to 12 months of age, child is defined as 1 year to pre-pubescent, and adult is defined as post-pubescent (Table 1). A glossary for other terms used can be found at the end of the manuscrip.t

**Table 1:** Recommendation Summary

Outcome/Recommendation The balance between desirable and undesirable consequences were uncertain for this reason we suggest consideration of this option:	Strength of Recommendation	Quality of Evidence	Key References
a) Activities of Daily Living Relating to Self-Care			
• All patients with subtypes of EB prone to contractures and decreased mobility should have an OT referral for clinical evaluation and assessment of their functional independence in ADL. (Additional file 1 and Additional file 2a)	D✓	3	[7–9]
• Patients should be an integral part of deciding therapy goals and the focus of OT intervention.	D✓	3	[10]
• Standardized assessments, checklists, and measures should be used to rate baseline ADL skills and change over time as well as monitor their functional status for any difficulties that may arise. (Additional file 2a)	D✓	4	Expert Opinion
• OTs are trained to assess a patient’s abilities to perform self- care activities and provide consultation regarding appropriate modifications, adaptations, and recommendations of equipment to aid independence. (Additional file 3)	D✓	4	[7, 11–15]
• Infant and child: Infants and children with EB should be encouraged to explore their environments, perform self-care, and participate in gross motor activities with efforts to minimize blister formation.	D✓	4	Expert Opinion
b) Instrumental Activities of Daily Living			
• OTs should use standardized assessment to identify baseline and progressive status of IADL and patients perceived QoL (Additional file 1 and Additional file 2a).	D✓	3	[16]
• OTs have a role in promoting a physically active, healthy lifestyle for patients of all ages.	C✓	1-	[17]*
• OTs should promote education, work, and social participation in the community.	D✓	3	[10]
• OTs should use modifications to promote greater independence in leisure activities and travel. (Additional file 4)	D✓	4	[18, 19]
• Adult: Adults with EB can work with a therapist or be referred to a driving instructor who specializes in adaptations for driving if there are physical concerns that limit access. (Additional file 3)	D✓	4	Expert Opinion
c) Maximization of Hand Function			
• Infant and child: Patients at risk of developing hand deformities such as those with RDEB should receive a hand evaluation within the first 1-2 years of life with regular monitoring of deformities.	D✓	4	[9, 20, 21]
• If hand involvement is observed, the OT should perform a thorough hand evaluation that includes measurements of web space/finger length, range of motion (ROM), and hand function. (Additional file 1)	D✓	4	[9, 11, 20–22]
• Regular monitoring of hand status should be provided.	D✓	4	Expert opinion
• OTs should provide home exercise programs to caregivers including daily active hand ROM exercises. This is particularly important for RDEB AND JEB subtypes. (Additional file 5)	D✓	4	[15, 20, 21, 23]
• For persons with EB who demonstrate the development of finger contractures and/or web creep, OT treatment intervention may include web preserving wrapping, individual finger wrapping, use of thermoplastic orthoses with or without silicone inserts, or silicone molds. See footnote★ (Additional file 6)	D✓	4	[9, 11, 13, 20, 21, 23]
d) Fine Motor Development and Fine Motor Retention:			
• OTs should provide standardized assessments and checklists for monitoring of fine motor skills for at risk patients (Additional file 2b).	D✓	4	[21, 22, 24, 25]^α^
• Infant and child: OTs should provide treatment intervention to facilitate the development of age appropriate fine motor skills and support social integration and improve QoL.	D✓	3	[9, 11, 21, 22]
e) Oral Feeding Skills (See Disclaimer in Box 1)			
• OTs should work closely with other MDT members involved with feeding including a dietician/nutritionist and speech and language therapist regarding the patient’s feeding needs.(refer to disclaimer)	D✓	4	Expert Opinion
• OTs may work with patients with EB to promote confidence with eating different food textures.	D✓	3	[19, 26]
• OT treatment intervention should include oral motor exercises.	D✓	4	[21]
• OTs should encourage the social components of eating during mealtimes regardless of use of alternative feeding methods (Naso-gastric or gastrostomy feeding tube) for integration of the patient into daily life and promote QoL.	D✓	4	[19]
• Child: Consider role of previous complications causing food aversions such as constipation and acid reflux in patient’s feeding presentation	D✓	4	[27]
Infant:			
➢ OTs may provide assessment of feeding in new-borns and babies and advise on modifications.	D✓	4	[13, 26]
➢ OTs should recommend optimal positioning to facilitate feeding skills.	D✓	4	Expert Opinion
➢ OTs should provide recommendations and consultation for multisensory and psychosocial components to the function of eating.	D✓	4	Expert Opinion
**Box 1. Disclaimer: In some countries OTs advise on feeding and swallowing skills. Review the practice act for the country of residence to ensure that this activity is within the scope of OT practice and if certification is required.**			

Key EB=epidermolysis bullosa; OT/OTs=occupational therapy/therapist; ADL=activities of daily living; IADL=instrumental activities of daily living; QoL=quality of life; RDEB= recessive dystrophic epidermolysis bullosa; MDT=multidisciplinary team; ROM= Range of Motion; Expert Opinion: This is the expert opinion of the panel members; * Articles where the sample population did not have epidermolysis bullosa; ^α^ Article 25 was not appraised due to being published past the appraisal period

a. Grades Recommended best practice based on the clinical experience of the guideline panel descriptions in accordance to SIGN [4]

D Theoretical/foundational-A preponderance of evidence from animal or cadaver studies from conceptual/theoretical models/principles, or from basic science/bench research, or published expert opinion in peer-reviewed journals supporting the recommendation. Evidence level 3 or 4; or Extrapolated evidence from studies rated as 2+

b. Rate Level Quality of evidence level descriptions in accordance to SIGN [4]

1- systematic review with a high risk of bias

3 Non-analytic studies, e.g. case reports, case series

4 Expert opinion

c. ✓ Recommended best practice based on the clinical experience of the guideline development group [4]

Notes ★ The use of hand wrapping and orthoses intervention to maximize hand function was discussed and delineated with the panel. Evidence gathered from appraised articles as well as expert opinion from the clinicians on the panel recommend these interventions. There was a difference of opinion by the panel member living with EB who has hand involvement and feels that wrapping may have caused his skin to be more fragile and that these interventions may have been of limited value in preventing web creep

The majority of the articles were graded level 3, being small-scale case studies, or level 4 for expert opinion, all recommendations were regarded as best practice based on the clinical experience of the guideline development group (Table 1).

The searches identified 70 articles, of these 56 articles were specific to the EB population. Post appraisal 30 articles were included and a final 27 articles were chosen for the final recommendations. The articles summary and qualities are outlined in Table 2.

**Table 2:** Overview of evidence per outcome

Outcome	Number of allocated papers	Total participants with EB *	Methodologies	SIGN rate ^ref^	Average quality rate % (range)	Benefits and limitations
Activities of Daily Living Relating to Self Care	**9**	3496* EBS 1856+ JEB 297+ DEB 486+ RDEB 505+ KS✓	2 NEBR 2 qualitative study 3 expert opinion ⇒1CG 1 consensus	**2-**[8] **3**[9] **3**[10] **3**[14] **4**[11] **4**[12] **4**[13] **4**[7] **4**[15]	**48** (19-75)	Six articles did not specify EB subtype numbers. Two studies are based on NEBR database of 3280 subjects. This affected the total number of participants. Three articles are expert opinion based on limited literature review. One article states numbers of children 140 to adults 234 and another Male 11 to Female 13.
Instrumental Activities of Daily Living	**5**	115* EBS 62+ JEB 8+ DEB 25+ RDEB 16+	1 qualitative study 1 quantitative study 1 systematic review 2 expert opinion	**3**[10] **3**[16] **1-**[17]^α^ **4**[18] **4**[19]	**55** (28-75)	Three articles did not specify EB subtype numbers. One was not specific to persons with EB. One states numbers of male 11 to female 13 participants. Two are expert opinion based on limited literature review.
Maximization of Hand Function	**8**	3351* EBS 1700+ JEB 247+ DEB 437+ RDEB 457+	1 NEBR 5 expert opinion 1 consensus 1 cross sectional	**3**[9] **4**[11] **4**[13] **4**[20] **4**[21] **4**[15] **3**[22] **4**[23]	**41** (19-71)	Six articles did not specify EB subtype numbers. One study used a database of 3280 subjects. One qualitative questionnaire study was carries out in children age 2-18 years old and had 39 girls to 32 boys’ participants. Four articles are expert opinion based on limited literature review.
Fine Motor Development and Fine Motor Retention	**5**	3403* EBS 1710+ JEB 247+ DEB 442+ RDEB 473+	1 NEBR 2 expert opinion 1 cross sectional 1 pilot study	**3**[9] **4**[11] **4**[21] **3**[22] **3**[24, 25]^β^	**54** (19-71)	Five articles did not specify EB subtype numbers. One study used the NEBR database (n=3280). One was a pilot study that has been recently validated, [25]^β^ but published post appraisal stage. This study was carried out in children (16) and adults (15); age 1-50 years old and had 14 females to 17 male participants. Two are expert opinion based on limited literature review.
Oral Feeding Skills	**5**	No values	5 Expert opinion	**4**[13] **4**[21] **4**[19] **4**[26] **4**[27]	**42** (28-53)	No articles specify EB subtype numbers. All articles were expert opinion, based on limited literature review.

Key: *total number of persons with EB in all papers combined; *Ref* references, *%* percentage, *EB* epidermolysis bullosa, *RDEB* recessive dystrophic epidermolysis bullosa, *JEB* Junctional epidermolysis bullosa, *DDEB* dominant dystrophic epidermolysis bullosa, *EBS* Epidermolysis bullosa simplex, *NEBR* National EB Registry, *KS* Kindler syndrome, +: value can be more as some papers did not state values, ✓: reported present but no value given, *CG* consensus guideline, n: number of, α: article not on EB population, β: Article 25 was not appraised due to being published past the appraisal period

## Recommendations

### ADL relating to self-care (strength of recommendation Grade: D)

#### Referral and assessment

We recommend that patients with all subtypes of EB with functional or biomechanical impairments including contractures and decreased mobility receive an early OT referral for assessment of their functional independence in ADL with frequent re-evaluation. (Table 1a; Additional file 1)

- ➢ Many EB patients develop musculoskeletal contractures including the hands and feet leading to further impairments in their abilities to perform basic ADL such as dressing, grooming, and bathing.
- ➢ Those with the more severe forms of EB such as persons with recessive DEB (RDEB) may have the greatest involvement and challenges, particularly if they spend prolonged periods in one position such as in a wheelchair.

OTs should use standardized assessments, checklists, and measures to rate baseline ADL skills and change over time (Table 1a; Additional file 2a) [28–33].

- ➢ The panel recognised the importance of a validated evaluation form to help standardise this process. An OT focused evaluation form was adapted by expert panel [7–9] (Table 1a; Additional file 1). This evaluation form will be piloted with the final CPG. For other age appropriate assessments forms see Additional file 2a.

Persons with EB are provided with modifications that are needed to limit cutaneous injury while enabling natural motor development, independence, and social integration that affects QoL (Table 1a; Additional file 3).

- ➢ Modifications to promote greater independence in ADL need to be integrated and accepted into the patient and family environment.
- ➢ OT consultation may include clothing choices or environmental modifications, adapting tools such as toothbrushes, and recommending equipment for bathing and toileting.
- ➢ Infant: Consultation and recommendations to parents of infants include [12–15]:
- Padding of bony prominences such as hips and elbows. An example is to use small kneepads as the baby begins to crawl. Baby sized kneepads or extra padding using dressings with tubular gauze to secure may be beneficial.
- Recommending clothing made of easy to slide material such as silk and using loose fitting clothing with front openings.
- Using disposable diapers lined with soft material to avoid friction.
- Handling the infant without causing unnecessary trauma such as lifting with one hand beneath the baby’s bottom and one behind the neck instead of the axillae. Or sliding hands below the mattress or using the sheet to lift and carry the baby.

#### Working in partnership

We recommend that patients should be an integral part of deciding therapy goals and the focus of OT intervention appropriate for their age and developmental level.

- ➢ OT intervention should be an individualized and patient centred collaboration. The patient should provide input in prioritizing areas of self-management and self-care in order to optimize independence [10] (Table 1a).
- ➢ OTs provide assessment of a patient’s abilities to perform self-care activities and provide consultation regarding appropriate modifications, adaptations, and recommendations of equipment to aid independence [7, 11–15, 30] (Table 1a; Additional file 3).
- ➢ Families of babies and toddlers with EB should be encouraged to allow their children to explore their environments, perform self-care, and participate in gross motor activities with efforts to minimize blister formation. This is important for the child’s overall development and learning to become more independent, although with greater activity, there may be more wounds. Encouraging independence, exploration, and involvement with activities is a life-long skill that needs to begin early.

⇒ Mobility, positioning, and positioning equipment are being addressed by the EB PT CPG (estimated date of publication 2019) and sexuality is being addressed by the EB Sexuality CPG (estimated date of publication 2020).

### Instrumental ADL (strength of recommendation Grade: D)

#### Assessment and modifications

We recommend that OTs should use standardized assessment tools and measurements to identify baseline and progressive status of IADL and patients’ perceived QoL (Table 1b; Additional file 1)

- ➢ The majority of common standardized assessment tools [28–30] and measurements have not been validated with the EB population (Additional file 2a).
- ➢ QoL in EB (QoLEB)-a self-reported assessment of the patient’s perceived QoL as it relates to various activities and relationships [16] (Additional file 2a).

OTs work to provide adaptations to optimize participation and success in work and school, which can lead to confidence and even more opportunities for leisure and social participation in those settings [10] (Table 1b).

We recommend offering adults with EB to work with an OT or driving instructor who specializes in adaptations for driving to enable access to this mode of transportation if there are physical concerns that limit access (Table 1b; (Additional file 3).

#### Working in partnership

We recommend OT’s promote physical activity for EB patients of all ages to prevent disability (Table 1b) [17].

- ➢ As part of their assessment OT’s should evaluate, modify and support the patient, family and environmental factors to promote physical activity (PA). PA plan need to be in line with MDT and match the individual and family needs, developmental levels of the patient, and preferences.
- ➢ OTs can advocate for and develop accessible and, flexible community based programs and consult with personnel who interact with the patient such as an employer, educator, or coach.

OTs work with patients and their families to provide modifications to promote greater independence in leisure activities and travel [18] (Table 1b; (Additional file 4).

- ➢ Child and adult: There are camp programs that are available to children with skin disorders including EB. Camp experiences can be a positive, and enriching experience. According to research, a positive outcome is that some of the campers report decreased feelings of isolation [18, 19] (Additional file 4).
- ➢ The MDT may have local resources for opportunities for children and adults with EB. These activities should be encouraged.

### Maximization of hand function (Strength of recommendation Grade: D)

This CPG details recommendations for non-surgical interventions to maintain and optimize the full movement and strength potential of hand joints (Table 1c). ⇒ Hand surgery and post-surgical rehabilitation recommendations, will be addressed by the Hand Surgery and Rehabilitation CPG (estimated date of publication 2020).

#### Early assessment and monitoring

We suggest consideration for patients at the greatest risk of developing hand deformities such as those with RDEB, JEB, and KS; a hand evaluation within the first 1–2 years of life with regular monitoring of deformities is recommended [9, 20, 21] (Table 1c).

OT’s should utilize a thorough hand evaluation form that includes web space/finger length measurements, finger range of motion (ROM) and assessments of hand function including the functions of grasp, pinch, and performance of ADL for at risk patients and those who have developed web creep and finger contractures [9, 11, 20–22, 34, 35] (Table 1c).

- ➢ The first web space between the thumb and index finger is of the greatest importance for maintaining the ability to pinch, grasp and write and needs to be assessed.
- ➢ A standardized assessment has not been validated for the EB population (Additional file 1)

Regular monitoring of hand status to be provided at least yearly and more frequently if there is contracture development and/or web creep.

OT’s should provide home exercise programs to caregivers including daily active and passive ROM for specific affected joints particularly if there is finger involvement and the use of recreational activities that involve body movement [15, 20, 21, 23, 30] (Table 1c; Additional file 5).

- ➢ Infant: OT must be started early in life in particular in generalized RDEB and JEB subtypes.
- ➢ The continuing work of muscles and joints may delay contractures and deformities, improve functional mobility, enhance patient autonomy, and, ultimately, promote social inclusion*.*
- ➢ For persons with EB who demonstrate the development of finger contractures and/or web creep, we recommend OT treatment intervention that may include individual finger wrapping, and the use of thermoplastic orthoses with or without silicone inserts [20] (Table 1c; Additional file 6).
- ➢ For patients with RDEB, preventative wrapping of individual fingers with tension in the web space, beginning in infancy may be recommended in an attempt to preserve function and attempt to delay fusing for as long as possible [9, 11, 13, 23].
- ➢ We suggest consideration of various methods of finger wrapping. These include wrapping to address web creep, as a dressing for finger wounds, and with force toward finger extension [36].
- ➢ The use of light, soft gloves that provide downward pressure between web spaces may be an adjunct or alternative to wrapping.
- ➢ Static (preventative) and dynamic (corrective) orthoses may be beneficial [9, 11, 21, 23]. The static orthosis is to be used primarily at night and the dynamic for periods of time during waking hours. Due to potential for wound skin breakdown, all recommended orthoses need to be monitored for proper fit and function.
- ➢ If the patient does not tolerate wrapping during the day, we suggest consideration of web preserving wrapping and/or use of an orthosis may be recommended to use at least at night.
- ➢ If the patient does wrap during the day, we suggest consideration of periods of time when they are free of wrapping to encourage somatosensory input and freedom of movement.

### Fine motor development and retention of Fine motor skills (Strength of recommendation Grade: D)

#### Assessment and monitoring

We recommend that OTs should provide standardized assessments of fine motor skill development and monitoring for at risk patients [21] (Table 1d; Additional file 2b).

OTs should provide treatment intervention to promote age appropriate motor development and support social integration [11, 20, 22, 30] (Table 1d).

- ➢ Research has found that better hand function was highly correlated with better reported quality of life for all of the subjects studied, with different types of EB.

OT’s monitor the progressive deformities of the hands and the impact this has to reduction in function, including reduced fine manipulative skills and loss of digital prehension [9] (Table 1d).

- ➢ OT recommendations may include modifications to improve the ability to perform fine motor tasks such as sheepskin used as modified grips, using soft ergonomic pens/pencils, and computers with a minimal touch mouse, touch screen, or speech recognition to be able to complete school work [11].
- ➢ OTs should work with persons with EB who have fine motor challenges on tasks involving bilateral hands and manipulative skills such as opening jars, buttoning trousers/pants, zipping up and snapping a jacket, and opening bags and screw top lids [22].

##### Special Considerations

The literature states that 5% of the general population has sensory processing deficits [37]. OTs should provide assessment and treatment of children with EB that demonstrate sensory processing deficits as this can impact fine motor development and skills. Interventions can address motor and perceptual development [11].

### Oral feeding skills (strength of recommendation Grade: D)

OT practitioners can provide essential services in the management of feeding, eating, and, in some countries swallowing conditions for people with a variety of EB diagnoses across the lifespan (Table 1e; Box 1).

#### Scope of practice and working in partnership

We recommend that OTs should work closely with other team members involved with feeding including a dietician/nutritionist, dentist, and speech and language pathologist/therapist regarding the patient’s feeding needs (Table 1e Box 1).

#### When appropriate and in line with scope of practice

We suggest consideration for OTs to monitor the patient’s feeding needs to promote confidence with eating different food textures (Table 1e)

- ➢ There may be previous *c*omplications causing food aversions such as oesophageal stricture, constipation and acid reflux [27] (Table 1e).
- ➢ Trying to limit stressful and protracted mealtimes to improve QoL [27].
- ➢ Encouraging older children to experiment with foods, providing individual guidance on suitable textures and taking into account food preferences. In some cases soft/pureed foods are encouraged; hot, acidic, spicy foods are discouraged [19].
- ➢ Everyone is an individual.

OT treatment intervention should consider inclusion of oral motor exercises when appropriate due to the risk of decreased jaw opening and tongue mobility [21]. (Table 1e; Box 1).

OTs should encourage the social components of eating during mealtimes regardless of use of alternative feeding methods (Naso-gastric or gastrostomy feeding tubes) for integration of the patient into daily life and promote QoL [19] (Table 1e).

- ➢ Infant and child: OTs should consider the role of previous complications causing food aversions such as constipation, anal fissures, and acid reflux in patient’s feeding presentation [27](Table 1e).
- ➢ Infant: OTs provide an assessment of feeding in new-borns and babies as needed and advice on appropriate modifications (Table 1e).
  - Using a MDT approach, OTs can promote breast feeding with babies with EB, including lubricating the nipple, introduce solids with soft, smooth edged spoon (Additional file 3), and progress textures/tastes at the child’s pace (Box 1).
  - Specialized teats/nipples may be required due to oral involvement [13] (Additional file 3).
  - If within the OT scope of practice, assess suck/swallow coordination for risk of aspiration (Box 1).
  - A specialized bottle may be useful to minimize trauma to the gum margin and control the flow for feeding, so that even a weak suck will allow satisfactory flow (Additional file 3).
  - Options to support eating solids may include use of soft shallow plastic spoon with rounded edges, parents’ fingertip, or from a piece of soft food. Foods containing lumps in liquid matrix are more difficult to control in the mouth and have the potential to increase negative feeding experiences. Force-feeding is counterproductive [26] and not recommended (Table 1d; Additional file 3).

OTs provide advice on optimal positioning to facilitate feeding skills (Table 1e).

- ➢ For instance, the caregiver may be instructed in the use of synthetic sheepskin or a soft sheet as a barrier to transfer and hold the baby during feeding.

OTs provide consultation and advice for multisensory and psychosocial components to the function of eating (Table 1e).

- ➢ These may include having the person with EB join in family meal times to allow engagement in the social interaction, and enable them to see, smell, and take tastes of the food.

## Conclusions

People with EB present with a spectrum of disabilities with many experiencing wounds with minor trauma that can lead to blister formation, scarring, pain, and contractures limiting their performance of activities of daily living within the home and community, and their full participation in the pursuit of personal goals. OT plays an important role in patient care as part of the MDT working with these patients and their families to maximize their functional abilities and improve their QoL. This CPG is intended to facilitate informed decision-making in the provision of OT services by clinicians. The strength of these recommendations is primarily based on case series, expert opinion, and one case controlled study in the literature. Collaboration of our panel of clinical experts was used to supplement and support the evidence. This guideline does not discuss all possible methods of care, and although it does provide some specific recommendations, the OT needs to collaborate with patients and their caregivers to clinically assess the appropriateness of a given intervention depending on the individual needs and circumstances and available resources.

Five clinical areas under OT management of patients with EB were identified by scoping survey and consensus of the panel to address in this CPG. OTs play a vital role as part of the MDT to optimize independence in ADL, IADL, self-feeding skills, fine motor development, and to maximize hand function.

Where OT is not available on a regular basis, the patient should work together with their care providers and healthcare team to determine goals to maximize independence. Examples of exercises, adaptive equipment, and orthoses are included in the additional files to enhance these recommendations and further assist in guiding those without access to OT.

### Further research

The authors acknowledge the limitations of high quality evidence based literature in patients with EB, requiring OT services. Thus, there is a need for further research to improve future care. Recommended areas for future studies include:

- Health Promoting Physical Activities, including Leisure and community activities, specific to EB population.
- Validity and Reliability of Standardized OT evaluation form for patients with EB.
- Effectiveness of hand orthoses and wrapping to improve hand function and fine motor skills.
- Effectiveness of modifications and adaptive equipment to promote functional independence.

### Updating procedure

It is anticipated that a literature search for new evidence pertaining to the provision of OT in EB will be undertaken every 3–5 years after publication in order to update the guidelines. These revised guidelines will be hosted by the DEBRA international website to ensure their availability and dissemination to clinicians, carers, and people with EB worldwide. The implementation of these recommendations could be monitored and evaluated through audits lead by DEBRA International (Additional file 7). The panel recommends sites to pre-audit practice, implement the CPG and re-audit to test improvement [4]. DEBRA International would value feedback on the site findings to continue to improve CPG quality.

### Implementation barriers

- Availability of resources (such as adaptive aides and hand orthoses)
- Limited and uneven distribution of knowledge and expertise.

## Additional files

Additional file 1: Occupational Therapy Evaluation for Epidermolysis Bullosa. (PDF 175 kb)

Additional file 2: Other a) assessment forms for OT and b) fine motor skills. (PDF 344 kb)

Additional file 3: ADL and IADL Equipment. (PDF 578 kb)

Additional file 4: International EB Camps and Activities Directory. (PDF 97 kb)

Additional file 5: Hand and Wrist Exercises. (PDF 460 kb)

Additional file 6: Orthoses. (PDF 334 kb)

Additional file 7: CPG Evaluation Form: Pre implementation. (PDF 139 kb)

## Abbreviations

ADL: Activities of Daily Living
AGREE: Appraisal of Guidelines for Research and Evaluation
AHP: Allied health professionals
CASP: Critical Appraisal Skills Programme
COPM: Canadian Occupational Performance Measure
COSA: Child Occupational Self-Assessment
CPG: clinical practice guideline
DEB: Dystrophic EB
DEBRA: Dystrophic epidermolysis bullosa association
EB: Epidermolysis bullosa
EBS: EB simplex
GRADE: Grades of Recommendation Assessment, Development, and Evaluation
HELP: Hawaii Early Learning Profile
ISCOR: Instrument for scoring clinical outcomes of research
JEB: Junctional EB
KS: Kindler syndrome
MDT: Multidisciplinary team
OT: Occupational therapist
PDMS: Peabody Developmental Motor Scales
PROMIS: Pediatric Reported Outcomes Measurement Information System
PT: Physiotherapist
QoL EB: Quality of Life in Epidermolysis Bullosa
QoL: Quality of Life
RDEB: Recessive Dystrophic epidermolysis bullosa
ROM: Range of motion
SIGN: Scottish Intercollegiate Guidelines Network
SP: Sensory Profile
SPM: Sensory Processing Measure
UK: United Kingdom
US: United States
WeeFIm: Functional Independence Measure for Children

## Glossary

ADL Relating to Self-care: Activities oriented toward taking care of one’s own body that are fundamental to living in a social world and enable basic survival and well-being. These include bathing and showering, toileting and toilet hygiene, dressing, eating/feeding, functional mobility, personal hygiene and grooming, personal device care and sexual activity
Instrumental ADL: Activities to support daily life within the home and community that often require more complex interactions than those used in self-care ADL. These include care of others, care of pets, child rearing, communication management, driving and community mobility, financial management, health management and maintenance, home establishment and management, meal prep and clean up, religious and spiritual activities, safety procedure and emergency response, shopping. As a panel, the areas of education, work, play, leisure, social participation, rest and sleep were included in this section as they are areas appropriate for adults
Hand Function: In this CPG hand function recommendations refer to non-surgical interventions to maintain and optimize the full movement and strength potential of hand joints to allow functional pinch, grasp, precision accuracy, coordination, and performance of ADL tasks
Fine Motor Development and Retention of Fine Motor Skills: Fine motor skills generally refer to one’s ability to control the small movements of the hands and fingers. Development of fine motor skills includes appropriate milestones in a child’s physical, mental, and behavioural development and includes the development of the sensory processing system. Sensory integration is the process by which we receive information through our senses, organize this information, and use it to participate in everyday activities
Oral Feeding Skills: Management of skills of oral feeding, eating, and, in some countries swallowing conditions for people with a variety of EB diagnoses across the lifespan
