# Supplementary material for: Occupational therapy for epidermolysis bullosa: clinical practice guidelines
Source: Orphanet J Rare Dis. 2019 Jun 7;14:129. doi: 10.1186/s13023-019-1059-8 (PMC6556021; doi:10.1186/s13023-019-1059-8)
Supplement: Supplementary file 1 — Occupational Therapy Evaluation for Epidermolysis Bullosa. (PDF 175 kb) [file 13023_2019_1059_MOESM1_ESM.pdf]

Occupational Therapy Clinical Practice Guideline: Additional file 1

Occupational Therapy Evaluation for Epidermolysis Bullosa

Client Information

**Name**		**Client ID**	
**Date of Birth**		**Evaluation Date**	
**Address**		**Phone Number**	
**Email**			
**Gender & Sex**			

EB/Medical History

**Type of EB**		**Primary Care Provider (PCP)**	
**Other Known Conditions or Diagnoses**		**PCP Address**	
**Medications**		**PCP Phone Number**	
**Dressing Materials**			
**Insurance/Payer**			
**Other Health Care Providers**			

Social History

**Family in Home**		**Primary Provider of Assistance in Home**	
**Relationship Status**		**Type of Home**	
**Sexual Orientation**		**Stairs In/At Home**	
**Caregiver Roles**			
**School**			
**Work**			
**Emotional Supports**			

Leisure and other Activities

	1 = Dependent	2 = A lot of Help	3 = Help with Half	4 = A little Help	5 = No Help Needed
**Leisure and other activities**	☐	☐	☐	☐	☐

Comments	

Occupational Therapy Clinical Practice Guideline: Additional file 1

Self-Care

	1 = Dependent	2 = A lot of Help	3 = Help with Half	4 = A little Help	5 = No Help Needed
**Dressing: Lower Body**	☐	☐	☐	☐	☐
Comments					
**Dressing: Upper Body**	☐	☐	☐	☐	☐
Comments					
**Grooming** (Hair, Teeth, Shaving, etc.)	☐	☐	☐	☐	☐
Comments					
**Eating (Including feeding tube use)**	☐	☐	☐	☐	☐
Comments					
**Bathing**	☐	☐	☐	☐	☐
Comments					
**Toileting**	☐	☐	☐	☐	☐
Comments					
**Removing/Applying Dressings/Bandages**	☐	☐	☐	☐	☐
Comments					
**Equipment used in home**					
**Equipment used outside of home**					
**Caregivers/Carers**					

Mobility

	1 = Dependent	2 = A lot of Help	3 = Help with Half	4 = A little Help	5 = No Help Needed
**Getting Around in the House**	☐	☐	☐	☐	☐
Comments					
**Negotiating Stairs (ascending/descending)**	☐	☐	☐	☐	☐
Comments					
**Getting Around School/Work**	☐	☐	☐	☐	☐
Comments					
**Getting Around Outdoors**	☐	☐	☐	☐	☐
Comments					
**Transportation in the Community**	☐	☐	☐	☐	☐
Comments					
**Flying and Distance Travel**	☐	☐	☐	☐	☐
Comments					
**Equipment used in home**					

Occupational Therapy Clinical Practice Guideline: Additional file 1

**Equipment used outside of home**	
**Caregivers/Carers**	

Sleep

**Equipment used in home**	
**Hours per night**	
**Difficulty falling asleep/staying asleep**	

Household Management

	1 = Dependent	2 = A lot of Help	3 = Help with Half	4 = A little Help	5 = No Help Needed
**Cleaning the Home**	☐	☐	☐	☐	☐
Comments					
**Cooking/Meal Preparation**	☐	☐	☐	☐	☐
Comments					
**Laundry**	☐	☐	☐	☐	☐
Comments					
**Opening Containers**	☐	☐	☐	☐	☐
Comments					
**Paying Bills/Managing Expenses**	☐	☐	☐	☐	☐
Comments					
**Environmental Controls/Appliances**	☐	☐	☐	☐	☐
Comments					

**Equipment used in home**	
**Caregivers/Carers**	

Work/School

	1 = Dependent	2 = A lot of Help	3 = Help with Half	4 = A little Help	5 = No Help Needed
**Writing**	☐	☐	☐	☐	☐
Comments					
**Typing**	☐	☐	☐	☐	☐
Comments					
**Reading**	☐	☐	☐	☐	☐
Comments					
**Working in Groups**	☐	☐	☐	☐	☐
Comments					
**Social/Professional Relationships**	☐	☐	☐	☐	☐

Comments					
**Participation in Physical Education**	☐	☐	☐	☐	☐
Comments					
**Adaptation/Modification Management**	☐	☐	☐	☐	☐
Comments					

**Equipment used in school/work**	
**Adaptations/Modifications**	
**Caregivers/Carers**	

Hand/Skin Assessment

**Hand Dominance**		
**Open or Blistered Areas**		
**Nature of Contractures**	(Flexion vs. Cocooning vs Web Space)	
**Gross Grasp**	Percentage remaining: Left ______%, Right _______%	
**Precision Pinches**<br>**(If limited, indicate level of function)**	<u>Left</u><br>Lateral Pinch – Full Function ☐ , Limited ☐ _______%<br>2-Finger Pinch – Full Function ☐ , Limited ☐ _______%<br>3-Finger Pinch – Full Function ☐ , Limited ☐ _______%	<u>Right</u><br>Lateral Pinch – Full Function ☐ , Limited ☐ _______%<br>2-Finger Pinch – Full Function ☐ , Limited ☐ _______%<br>3-Finger Pinch – Full Function ☐ , Limited ☐ _______%

Functional Digital Use

Task	Pinch Utilized<br>(Lateral, Pincer, 3-Jaw, Bimanual, Other)	Level of Independence<br>(Independent, Modified – Uses equipment or adaptation, Dependent – Requires assistance)
Turning Key		
Picking Up a Coin		
Grasping a Pencil		
Finger Feeding		
Fork/Spoon Use		
Knife Use		
Holding a Cup		
Using Scissors		
Grasping Shoe String		
Other (Indicate task):		

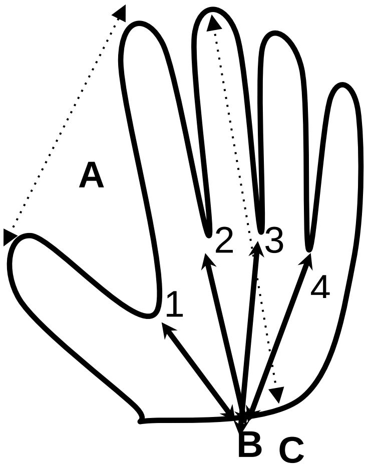

- Draw an imaginary line connecting the ulnar styloid and radial head.
- B-Web spaces are measured from the imaginary line ~~between~~ at the wrist to the end of the web spaces.
- The length of the middle finger is measured. (C)
- Monitor web creep by comparing the change in long finger length with web length to account for normal growth.

Reference: Lipnick et al (1992) Physical rehabilitation of epidermolysis bullosa patients; Epidermolysis bullosa: Basic and clinical aspects. Weiss and Prinz (2013) also describe a method of web space measurement. In addition, various clinicians that work with persons with EB have developed, but not published, alternative methods.

	**Left**				**Right**			
**A. First web opening**								
**B. Wrist to web**	**1**	**2**	**3**	**4**	**1**	**2**	**3**	**4**
**C. Wrist to tip of middle finger**								

Evaluation Summary

ADDITIONAL COMMENTS

GOALS
(as agreed upon by client and team)

FOLLOW UP/PLAN OF CARE
