# Supplementary material for: Occupational therapy for epidermolysis bullosa: clinical practice guidelines
Source: Orphanet J Rare Dis. 2019 Jun 7;14:129. doi: 10.1186/s13023-019-1059-8 (PMC6556021; doi:10.1186/s13023-019-1059-8)
Supplement: Supplementary file 2 — Other a) assessment forms for OT and b) fine motor skills. (PDF 344 kb) [file 13023_2019_1059_MOESM2_ESM.pdf]

Occupational Therapy Guideline: Additional file 2

A. Assessment form	Validated for the EB population	Age range<br>**Infant**<br>(0-12 months)	 **Child**<br>(1 year - pre-pubescent)	 **Adult**<br>(post-pubescent)	Benefits	Limitations
**Quality of Life Evaluation in Epidermolysis Bullosa (QOLEB)**[16]	**Validated on patients with EB**	No	Yes	Yes	Useful for all EB subtypes	Children under 10 may need caregiver assistance to complete
**Child Occupational Self-Assessment (COSA)**[29]	Not validated on patients with EB	No	6-17 years	No	A self-report of occupational competence and value for everyday activities that uses visual cues for rating	Can be used for younger children depending on their maturity or with caregiver assistance as needed.
**COSA adapted for persons with EB**[30]					a modified version for children with EB	
**Pediatric Reported Outcomes Measurement Information System (PROMIS)**[31]	Not validated on patients with EB	No	8-17 years	Yes	An NIH supported group of self-report scales that include upper extremity skills and activities of daily living that can be web-based	Developed in English and research of validity and reliability performed in the United States
**Functional Independence Measure for**	Not validated on patients with EB	Yes	Yes	No	An assessment using observation and interview on 18	More difficult to rate younger children 2-4 years

**Children (WeeFim)**[32]					items to measure functional performance in self-care, mobility, and cognition.	
**Hawaii Early Learning Profile (HELP)**[33]	Not validated on patients with EB	Yes	0-6 years	No	A checklist designed to assess function in 6 areas of development including fine and gross motor skills and adaptive/self-help areas Translated into 8 languages	No peer-reviewed publications evaluating the reliability or validity were located

**B. Standardized assessments of fine motor skill**	**Validated for the EB population**	**Age range** Infant (0-12 months)	Child (1 year - pre-pubescent)	Adult (post-pubescent)	**Benefits**	**Limitations**
**Iscor EB** [24]	**Validated in children with EB**	No	1-17 years	18- 50 years	A questionnaire developed for persons with EB includes use of hands as an outcome in their assessment[24]	There is a more recent reference for the Iscor EB[25], published in 2018 that was not appraised as publication occurred after appraisal process for OT CPG was completed[25]
**Abilhand-Kid**[22]	**Validated in children with EB**	No	6-15 years	No	A questionnaire assessing manual dexterity, upper extremity function and various ADL functions	Based on patient and caregiver perceived difficulties
**Abilhand-adults**[37]	Not validated on patients with EB	No	No	16 -80 years		
**Patient Reported Outcomes Measure (PROMIS)**[38]	Not validated on patients with EB	No	Adolescent to young adult	Yes	An NIH supported group of self-report scales that include upper extremity skills and activities of daily living that can be web-based for adults	Validity and reliability studies on young adults

**Sensory Profile (SP)**[39]	Not validated on patients with EB	No	5-10 years	No	A group of questionnaires with three main sections; sensory processing modulation, and behavioural and emotional responses	used primarily by English speaking countries
**Sensory Processing Measure (SPM)**[39]	Not validated on patients with EB	No	5-12 years	No	A questionnaire to be filled out by caregivers, main classroom teacher, and other school personnel assessing social participation, praxis, and sensory processing issues	Used primarily by English speaking countries
**Peabody Developmental Motor Scales (PDMS)**[40]	Not validated on patients with EB	Yes	Birth-71 months	No	Fine motor subtests provide objective information to supplement the entire evaluation and support the need for therapy services	May not be sensitive enough to detect deficits with mild impairment

References here and not from the main body of text
37. Simone A, Rota V, Tesio L, Perucca L. Generic ABILHAND questionnaire can measure manual ability across a variety of motor impairments. Int J Rehabil Res. 2011;34:131-140.
38. Howell C, Gross H, Reeve B, DeWalt D, Huang I. Known-groups validity of the Patient-Reported Outcomes Measurement Information System (PROMIS) in adolescents and young adults with special healthcare needs. Qual Life Res. 2016;25:1815-1823.

39. Brown T, Morrison I, Stagnitti K. The convergent validity of two sensory processing scales used with school-age children: Comparing the Sensory Profile and the Sensory Processing Measure. N Z J Occup Ther. 2010;57:56-65.
40. Van Hartingsveldt MJ, Cup EH, Oostendorp RA. Reliability and validity of the fine motor scale of the Peabody Developmental Motor Scales–2. Occup Ther Int. 2005;12:1–13.
