# Supplementary material for: Occupational therapy for epidermolysis bullosa: clinical practice guidelines
Source: Orphanet J Rare Dis. 2019 Jun 7;14:129. doi: 10.1186/s13023-019-1059-8 (PMC6556021; doi:10.1186/s13023-019-1059-8)
Supplement: Supplementary file 3 — ADL and IADL Equipment. (PDF 578 kb) [file 13023_2019_1059_MOESM3_ESM.pdf]

ADL and IADL Equipment

Product	Geographical location	Estimated cost in US $
**Travel for babies and children**		
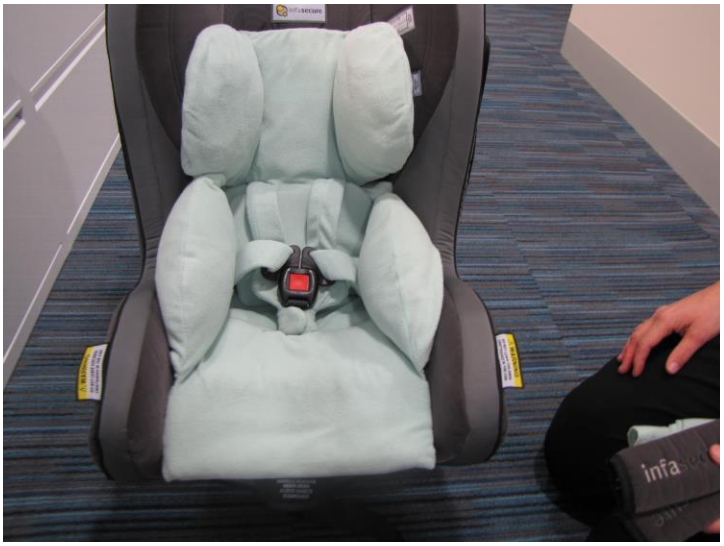<br>**Figure 1:** Car seat with plush covering	Norway, US, Mexico<br>DISCLAIMER<br>CHECK MANUFACTURER'S SPECIFICATIONS REGARDING THE ADDITION OF INSERTS AND SAFETY REGULATIONS FOR THE COUNTRY YOU LEAVE IN	15-27.00 for insert.<br>90-200.00 for padded car seat.
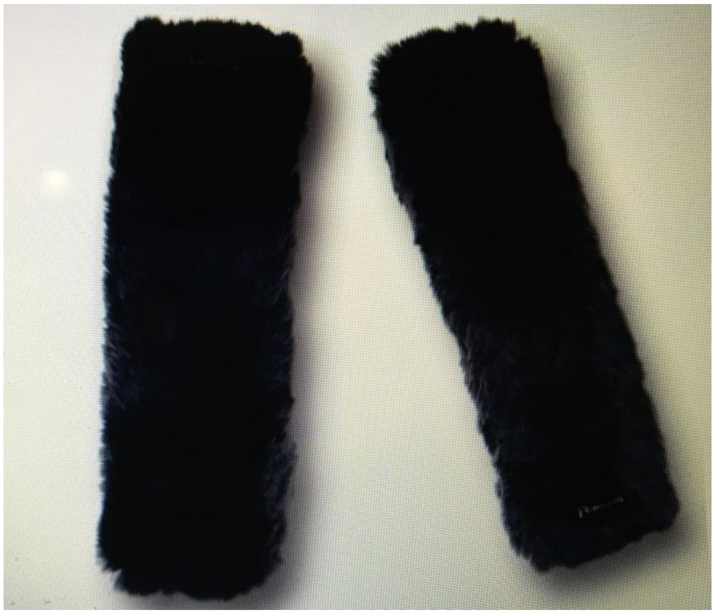<br>**Figure 2:** Car seat strap covers	Norway, US, and Mexico	8-13.00

Product	Geographical location	Estimated cost in US $
**Feeding Babies**		
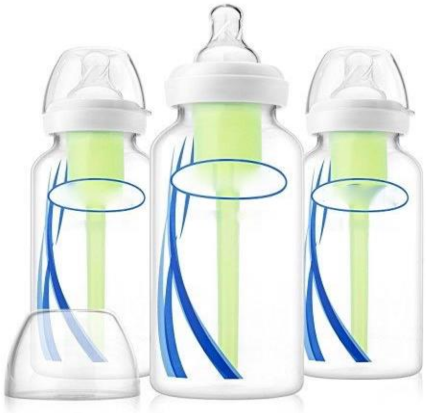 **Figure 3a:** Baby bottle with controlled flow	Norway, US, Mexico	16-23.00
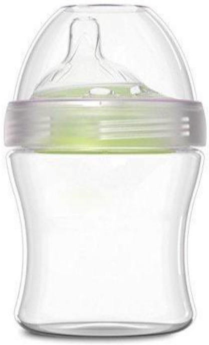 **Figure 3b:** Baby bottle with controlled flow	Norway, US, Mexico	17-20.00
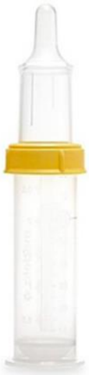 **Figure 3c:** Baby bottle with controlled flow	Norway, US, Mexico	23-30.00

Product	Geographical location	Estimated cost in US $
**Feeding tools**		
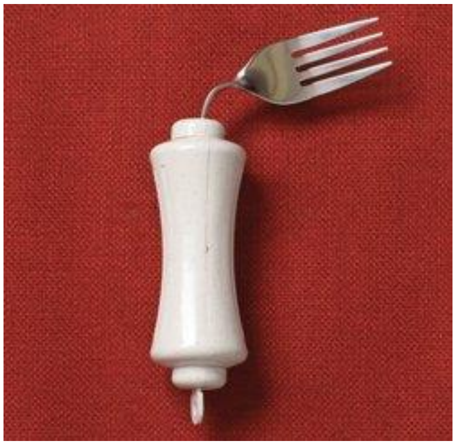 **Figure 4:** Adapted fork with built-up handle and curved to decrease range of motion needed to self-feed	Norway, US, Mexico	10-13.00
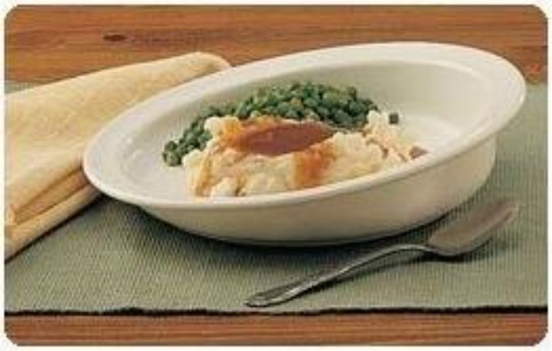 **Figure 5:** Scoop dish	UK, US, Australia	8-12.00
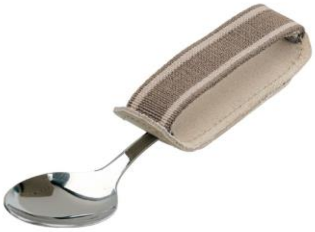 **Figure 6:** Adapted spoon for self-feeding	Norway, US, Mexico	9-21.00

Product	Geographical location	Estimated cost
**Gripping tools**		
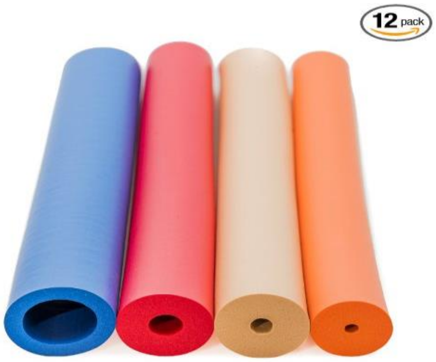<br><br>**Figure 7a:** Foam tubing to adapt handle size for ease of grip	Norway, US, and Mexico	10-19.00 for set of 3
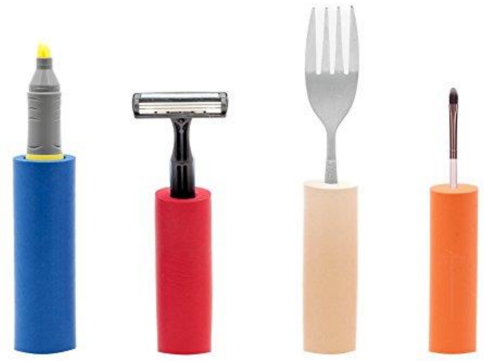<br>**Figure 7b:** Examples of use of foam tubing		
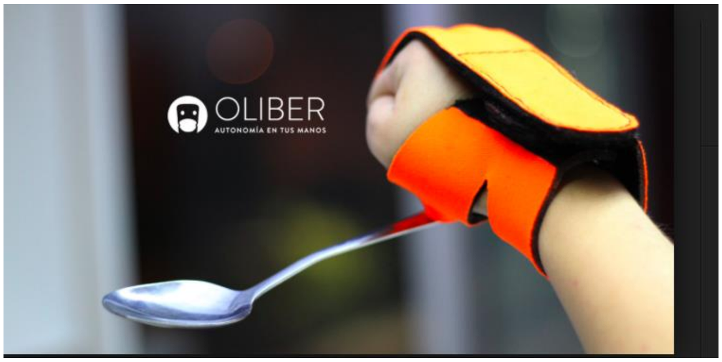<br><br>**Figure 8:** Oliber magnetic hand orthoses	Chile, USA, UK	www.myoliber .com

Product	Geographical location	Estimated cost
**Pads and socks**		
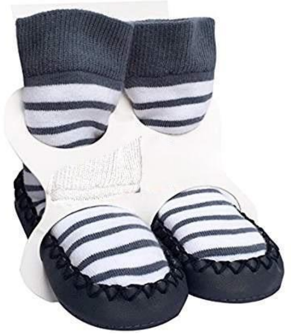<br>**Figure 9:** Baby slipper socks	Norway, US, Mexico	5-14.00
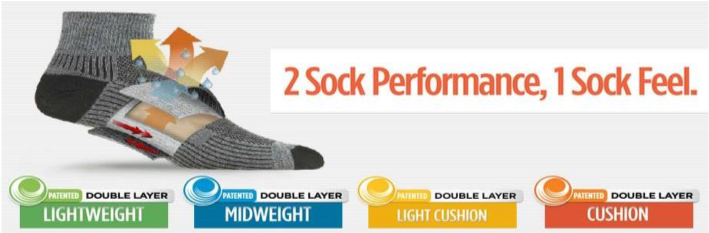<br><br>**Figure 10:** Double-layer anti-blister socks	Norway, US, Mexico	9-28.00
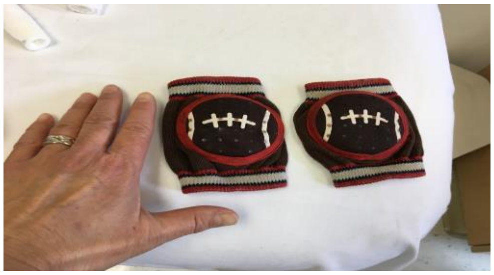<br>**Figure 11:** Baby-size knee pads	Norway, US, Mexico	9-12.00
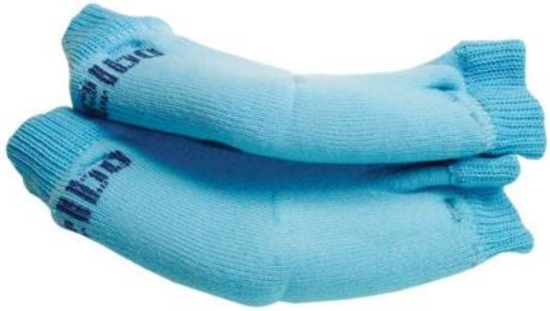<br>**Figure 12:** Padded elbow sleeves	Norway, US, Mexico	10-14.00

Product	Geographical location	Estimated cost
**Bath seats and handling aids**		
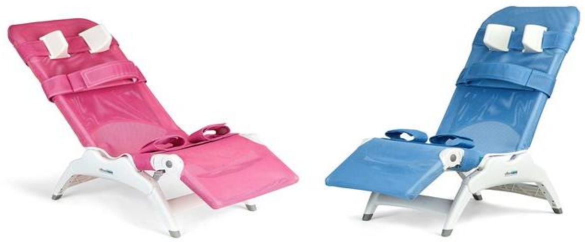<br>**Figure 13:** Mesh bath seats	Norway, US, Mexico	140-486.00
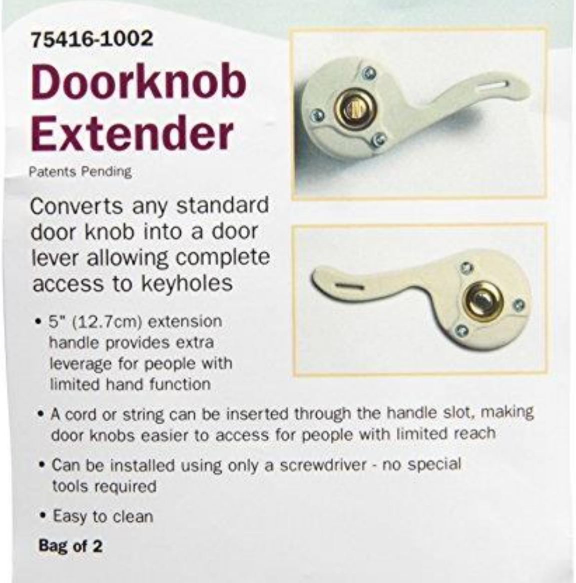<br>**Figure 14:** Door knob aid	Norway, US, Mexico	12-30.00
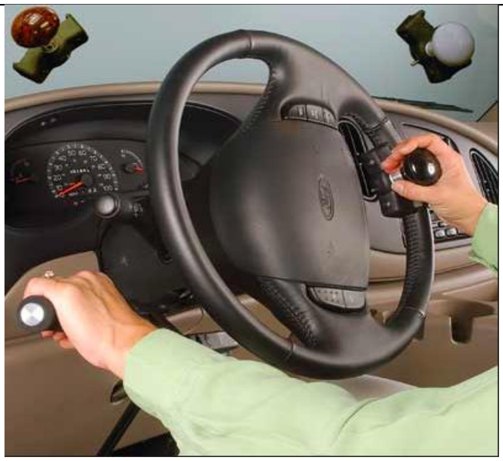<br>**Figure 15:** Steering wheel aid	Norway, US, Mexico	9-15.00

Product	Geographical location	Estimated cost
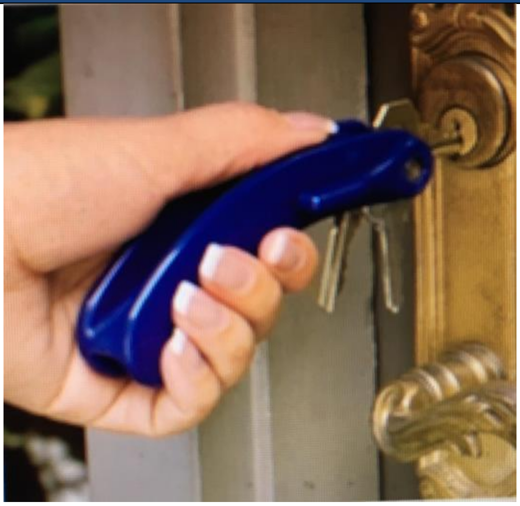<br>**Figure 16:** Key grip aid	Norway, US, Mexico	7-10.00
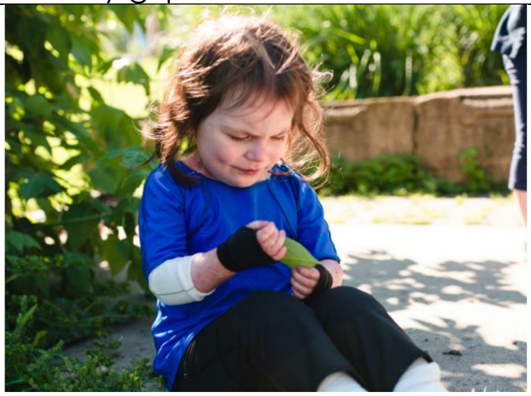<br>**Figure 17:** Glidewear is a manufacturer of ultra-low-friction fabric used for clothing, blankets, pillow covers and other accessories for persons with EB. It is a two-layer fabric that protects skin by gliding smoothly against itself and absorbing the harmful friction and shear that can damage skin.	US and UK	https://glidewear.com/

NOTE: For more recommendations, aids, and suggestions for ADL and IADL equipment, please refer to Occupational Therapy in Epidermolysis Bullosa by Weiβ and Prinz (2013) and the DEBRA International website (www.debra.org).
