# Supplementary material for: Occupational therapy for epidermolysis bullosa: clinical practice guidelines
Source: Orphanet J Rare Dis. 2019 Jun 7;14:129. doi: 10.1186/s13023-019-1059-8 (PMC6556021; doi:10.1186/s13023-019-1059-8)
Supplement: Supplementary file 4 — International EB Camps and Activities Directory. (PDF 97 kb) [file 13023_2019_1059_MOESM4_ESM.pdf]

International EB Camps and Activities Directory

Host	Name of the activity	Type of activity	Target groups	Location	Contact
**DEBRA of America**					
	**debra Care Conference**	This is a multi-day event made specifically for EB families and the professional community to access the best information, discover new ideas and connect with other EB families from all over the country **Different rate packages for EB families, non-EB families, professionals.**	EB families and the professional community	Location changes in USA	http://www.debra.org/debra-hosted-events
**DEBRA Argentina**					
	**Occupational therapy (OT) service**	OT service only at the DEBRA House, not outreach service. In Spanish only	All ages and family	DEBRA House	www.debraargentina.org
**DEBRA Australia**					
	**Kids Camp and Conference**	Every 2-3 years this is a great opportunity to network with other families and learn all things EB. The children have an amazing time and make lifelong friendships. **Registration fee has been heavily subsidised by DEBRA**	Families living with EB, and any extended family	Location changes in Australia	https://www.debra.org.au/family-support/national-eb-camp/

Host	Name of the activity	Type of activity	Target groups	Location	Contact
**DEBRA Austria**					
*	**Family weekend**	Time out of daily life, exchanges of experiences. Main language is German, members also from Italy. **No fee to attend to members only.**	All ages and family	Location Ainring in Germany	Sabine Wittmann, DSA Sabine.Wittmann@debra-austria.org 0043/1/8764030-0
	**Annual DEBRA Austria Meeting**	Trainings for people with EB and their relatives; Social program; Exchange of experiences. Main language is German, members also from Italy and Germany. **DEBRA Austria pays most of the expenses (only the drinks at meals have to be paid by the members).**	All ages, members and their families	Salzburg Austria	Sabine Wittmann, DSA Sabine.Wittmann@debra-austria.org 0043/1/8764030-0
**DEBRA Chile**					
	**Holiday Break in the national country**	A group of thirteen EB patients' families are invited with everything included to a week of holidays in La Laguna beach. 41 people. **DEBRA Chile absorb all the costs for members only**	All ages and their families	Región de Valparaíso, La Laguna, Chile	Ana María Gonzalez amgonzalez@debrachile.cl +56993386091

Host	Name of the activity	Type of activity	Target groups	Location	Contact
**DEBRA Germany**					
	**Annual Weekend Families**	Family-feel with workshops and fun events for children	EB families with young children	Location changes in Germany	ieb@ieb-debra.de www.ieb-debra.de
	**Annual Weekend for Mothers**	Respite for mothers	Mothers with children with EB	Location changes in Germany	ieb@ieb-debra.de www.ieb-debra.de
**DEBRA New Zealand**					
*	**Adventure/snow camps**	The camps are run approximately every 2 or 3 years, depending on who is interested in organising and how many are interested in attending.	People with EB	Location changes in New Zealand	debra@debra.org.nz
	**Family camps**	Emphasis on fun family activities.	EB families with young children	Location changes in New Zealand	debra@debra.org.nz
**DEBRA Slovakia**					
	**Holiday Break Abroad**	Patients and their families go on one week vacation by the sea to heal the wounds and the skin. Not every year depending on who is interested and its members only.	All ages and their families	2014 Spain 2015 Croatia 2016 Bulgaria	http://www.debra-slovakia.org/ Kristína Ficeriová kristina.ficeriova@gmail.com +421911793945

Host	Name of the activity	Type of activity	Target groups	Location	Contact
**DEBRA Slovakia**					
	**Camp**	Patients and families meet annually to be informed about the last year's activities, presenting new materials for EB patients, latest information about EB research, sharing experiences. For members only and the organisation absorb the costs of accommodation, catering, meeting activities	All ages and their families	Slovakia<br>Every year somewhere else	Kristína Ficeriová<br>kristina.ficeriova@gmail.com<br>+421911793945
**DEBRA Spain**					
✱	**Annual DEBRA Spain Family Meeting**	It includes talks and workshops and activities for people with all types of EB and all ages. Main language is Spanish.<br>**It is available to anyone. Members of DEBRA Spain have a grant to attend and people from outside must pay their expenses (hotel and meals).**	All ages and their families	Madrid Spain	Evanina de Morcillo Makow<br>info@debra.es<br>00 34 952816434

Host	Name of the activity	Type of activity	Target groups	Location	Contact
**DEBRA Sweden**					
	**Camp**	Camp for all members annually	All ages and their families	location, activity and time of year varies	www.ebforeningen.se
**DEBRA United Kingdom (UK)**					
	**Annual Overnight Weekend**	Family-feel with workshops and fun events for children	All ages	Location changes in UK	Helen Weaver helen.weaver@debra.org.uk
	**Supported Weekend City Break**	Encourage independent living, peer-support and networking with medical and peers	Adults with severe EB	Location changes in UK	Helen Weaver helen.weaver@debra.org.uk
	**Four holiday homes**	Holiday homes adapted for use by people with EB	All ages	UK	Helen Weaver helen.weaver@debra.org.uk

Key: * The activity welcomes people from other countries, do check their conditions.

Others none-DEBRA EB Camps and Activities

Host	Name of the activity	Type of activity	Target groups	Location	Contact
**Brazil**					
	**Dermacamp**	Camp for children with skin diseases, they have large experience with EB	Children with skin diseases	São Paulo, Brazil	http://dermacamp.org.br
**United States**					
	**SeriousFun Camps**	Host family weekends and week-long kids-only summer camps and typically have medical/nursing staff available for both.	Family and kids-only	Location changes in USA	https://www.seriousfunnetwork.org/
	**The Center for Courageous Kids in Kentucky**	Similarly to the SeriousFun Camps but is independent of that organization.	Family and kids-only	Kentucky USA	http://www.courageouskids.org/
	**Camp Discovery** Program by The American Academy of Dermatology's (AAD)	Under the expert care of dermatologists and nurses, Camp Discovery gives campers the opportunity to spend a week with other young people with skin conditions having fun. **No fee to attend.**	Children ages 8-16 who have a chronic skin disease	Location changes in USA	https://www.aad.org/public/kids/camp-discovery

Host	Name of the activity	Type of activity	Target groups	Location	Contact
**United States**					
	**Camp Spirit**	The Colorado Winter Adventure Camp provides opportunity to enjoy winter activities in a safe environment. **No fee to attend.**	Children ages 9-18 with Recessive Dystrophic EB (RDEB)	Colorado USA	https://campspiritcolorado.org/
	**Camp Wonder** Program by The Children's Skin Disease Foundation's	This is dedicated to providing a safe and nurturing environment to children with a skin disease. **No fee to attend.**	children ages 7-16 living with a skin disease	Location changes in USA	https://www.csdf.org/camp-wonder

Key: * The activity welcomes people from other countries, do check their conditions.
