# Supplementary material for: Occupational therapy for epidermolysis bullosa: clinical practice guidelines
Source: Orphanet J Rare Dis. 2019 Jun 7;14:129. doi: 10.1186/s13023-019-1059-8 (PMC6556021; doi:10.1186/s13023-019-1059-8)
Supplement: Supplementary file 5 — Hand and Wrist Exercises. (PDF 460 kb) [file 13023_2019_1059_MOESM5_ESM.pdf]

Occupational Therapy Guideline: Additional file 5

Hand and Wrist Exercises.

Type of exercise	Instruction and how many repeats	How often per week
**With no tools**		
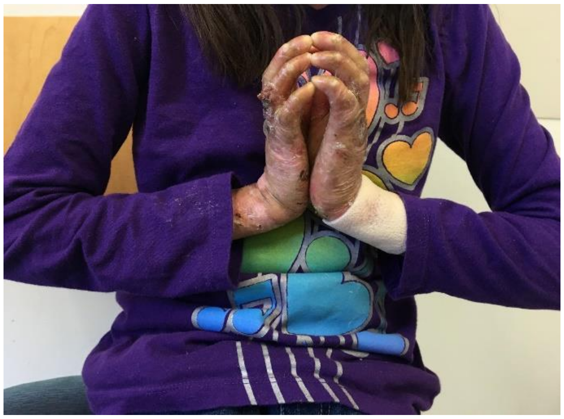<br>**Figure 1:** Wrist extension stretch.	Hold for 20-30 seconds and then repeat.	To be done daily.
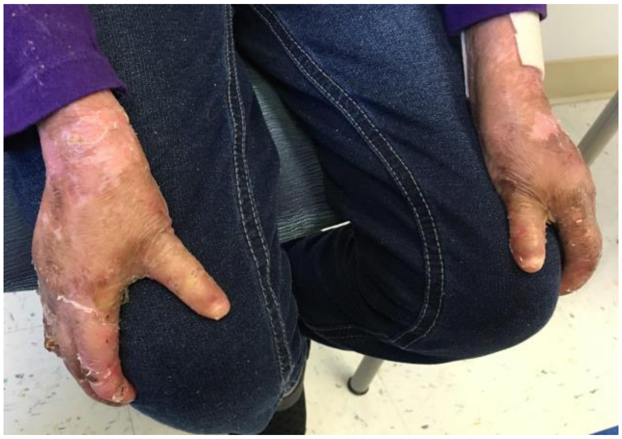<br>**Figure 2:** First web space stretch.	Stretch from the base of the thumb and hold for 20-30 seconds and repeat	To be done daily.
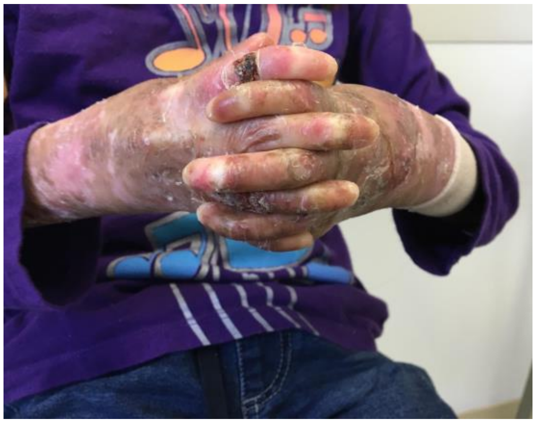<br>**Figure 3:** Interdigital finger stretching.	Hold for 20-30 seconds and repeat.	To be done daily.

Type of exercise	Instruction and how many repeats	How often per week
**With tools**		
a. b. 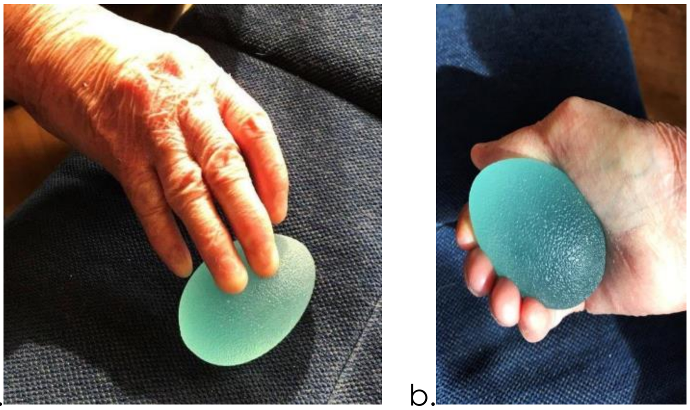<br><br>**Figure 4:** Hand exercises using a gel egg.	Roll the egg with fingers back and forth to stretch fingers into extension 10 times slowly<br><br>Grasp egg to gently stretch fingers and thumb open. Also stretch the base of the thumb. Hold for up to1-2 minutes.	To be done daily.<br><br>To be done daily.
a. b. 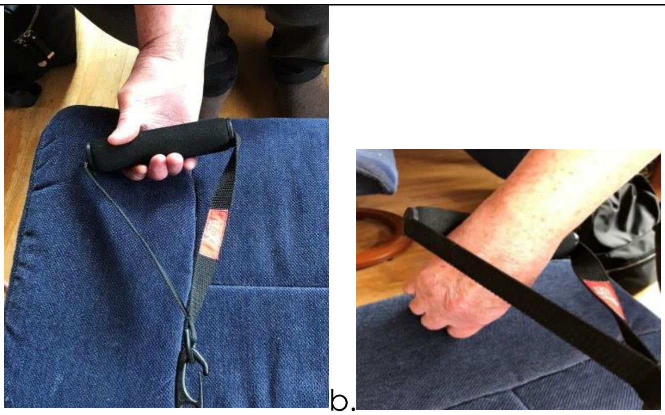<br><br>**Figure 5:** Finger and wrist exercises using a padded handle against light resistance.	Grasp handle and pull gently to stretch fingers into extension. Hold for a count of 10.	To be done daily.

**Type of exercise**	**Instruction and how many repeats**	**How often per week**
a. 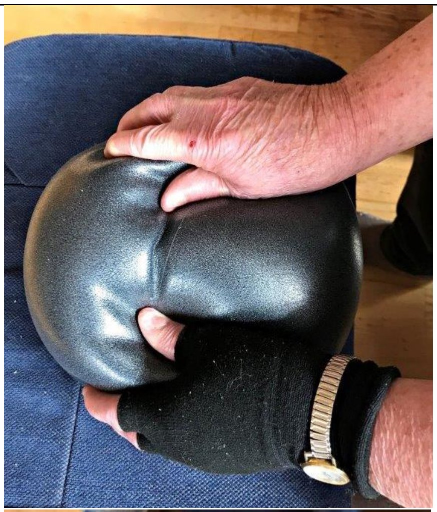	Gently grasp the ball, keeping the palm in contact with the surface. Use the ball to gently stretch the base of the thumb open. Hold for a slow count of 10, repeat.	This can be alternated with the stretch on the knee or the egg exercise above.
b. 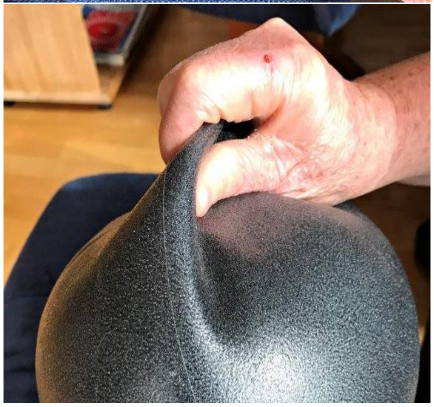 **Figure 6:** Hand exercise using a light, pliable ball for first web-space stretch and pinch strengthening.	Grasp and/or pinch the ball with fingers and thumb to maintain hand strength. Repeat 10 times.	To be done 3-5 times per week if it does not cause skin breakdown.

**Product**	**Geographical location**	**Estimated cost in US$**
Gel Egg	Norway, US, Mexico	6.00-9.00
Padded handle against light resistance	Norway, US, Mexico	2.00-6.00 to make, 12-15.00 to buy
Pliable ball	Norway, US, Mexico	8.00-12.00

For additional examples, resources and suggestions, please refer to Occupational Therapy in Epidermolysis Bullosa by Weiβ and Prinz (2013).
