# Supplementary material for: Occupational therapy for epidermolysis bullosa: clinical practice guidelines
Source: Orphanet J Rare Dis. 2019 Jun 7;14:129. doi: 10.1186/s13023-019-1059-8 (PMC6556021; doi:10.1186/s13023-019-1059-8)
Supplement: Supplementary file 6 — Orthoses. (PDF 334 kb) [file 13023_2019_1059_MOESM6_ESM.pdf]

Orthoses

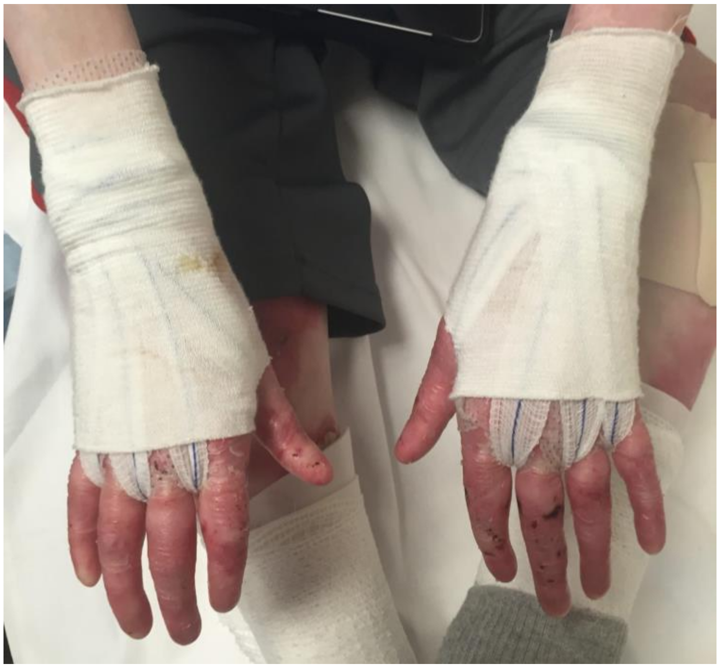

Figure 1: Interdigital finger wrapping

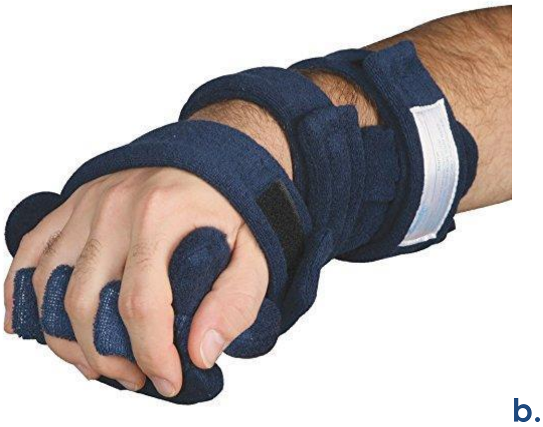

a.

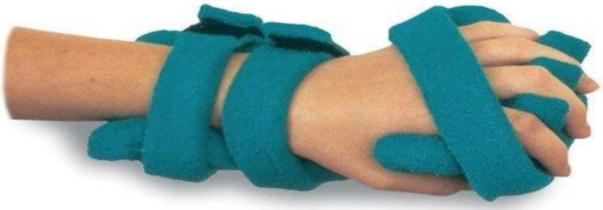

b.

Figure 2: Soft wrist and finger orthosis for **a.** night use **b.** shorter version

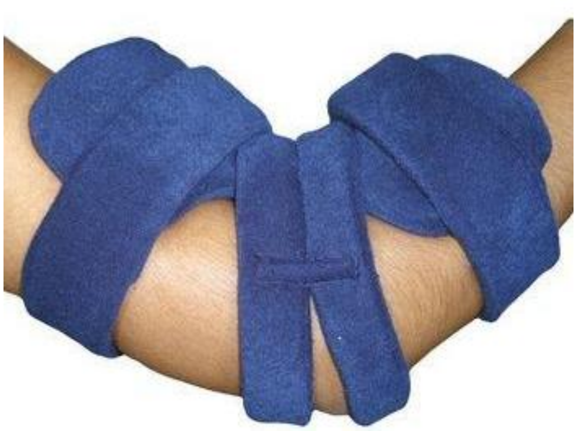

Figure 3: Soft elbow orthosis to maintain elbow extension

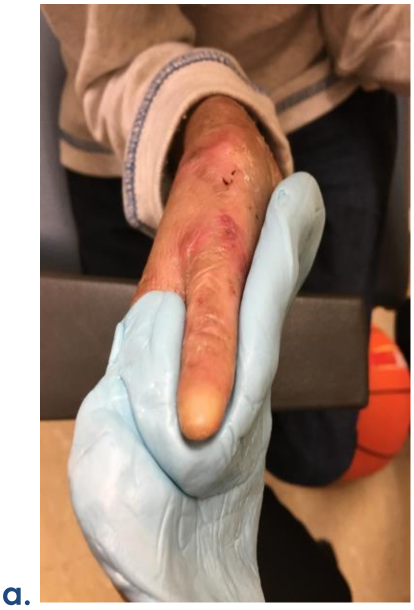

a.

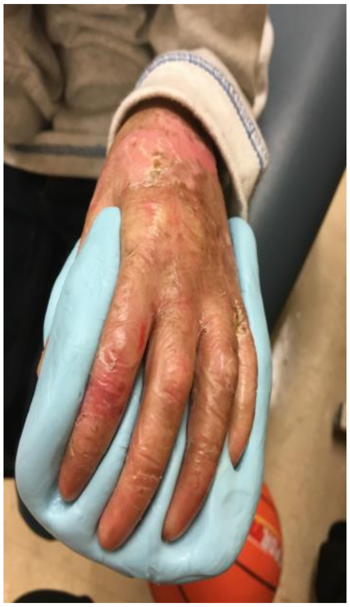

b.

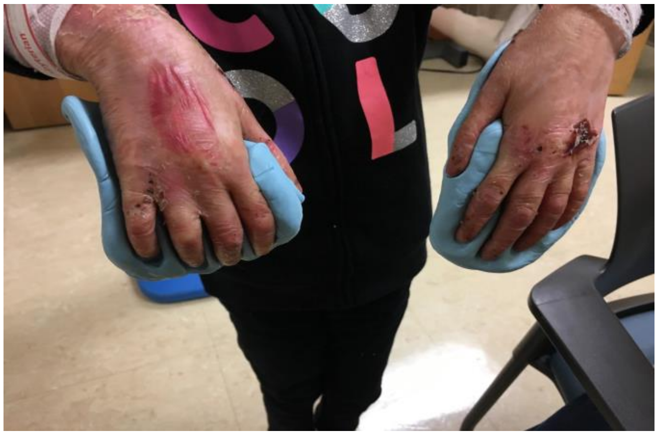

c.

Figure 4: Elastomer (silicone putty) **a.** palmar view; **b.** dorsal view; **c.** to prevent further web creep

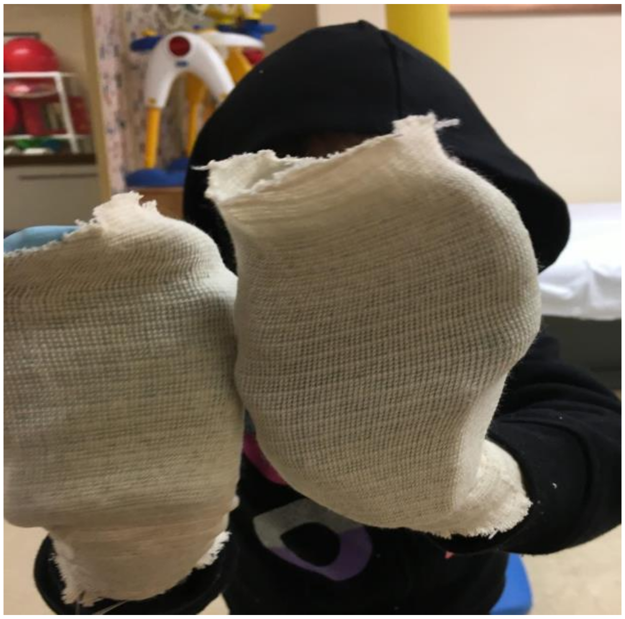

Figure 5: Elastomer orthoses secured with compressive sock material

a.

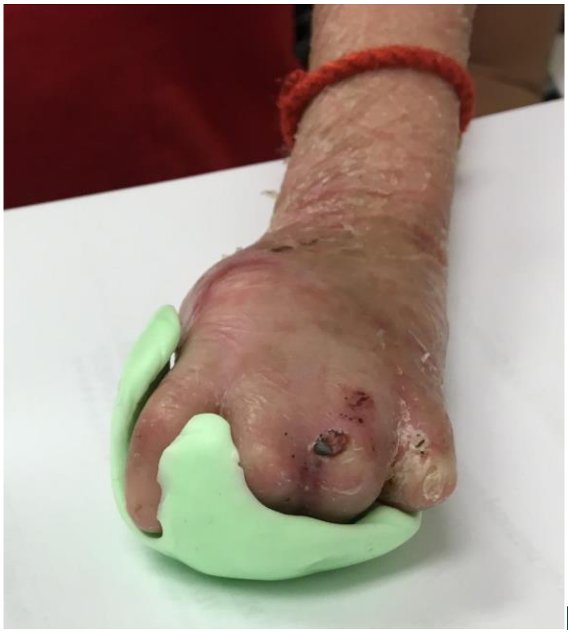

b.

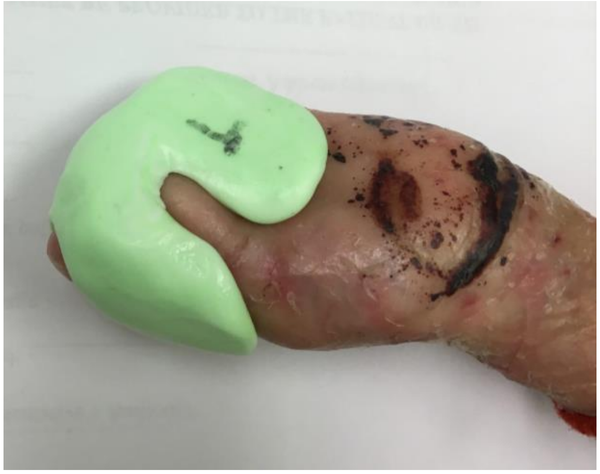

Figure 6: Elastomer used for **a.** maintenance of first web space, dorsal view; **b.** maintenance of first web space, palmar view

For additional examples, resources and suggestions, please refer to Occupational Therapy in Epidermolysis Bullosa by Weiβ and Prinz (2013).
